# HLA-A, HSPA5, IGFBP5 and PSMA2 Are Restriction Factors for Zika Virus Growth in Astrocytic Cells

**DOI:** 10.3390/v15010097

**Published:** 2022-12-29

**Authors:** Affan A. Sher, Ying Tenny Lao, Kevin M. Coombs

**Affiliations:** 1 Department of Medical Microbiology & Infectious Diseases, University of Manitoba, Winnipeg, MB R3E 0J9, Canada; 2 Manitoba Centre for Proteomics & Systems Biology, University of Manitoba, Winnipeg, MB R3E 3P4, Canada; 3 Children’s Hospital Research Institute of Manitoba, Winnipeg, MB R3E 3P4, Canada

**Keywords:** Zika virus, mass spectrometry, restriction factors, siRNA-mediated KD

## Abstract

(1) Background: Zika virus (ZIKV), an arbo-flavivirus, is transmitted via *Aeges aegyptii* mosquitoes Following its major outbreaks in 2013, 2014 and 2016, WHO declared it a Public Health Emergency of International Concern. Symptoms of ZIKV infection include acute fever, conjunctivitis, headache, muscle & joint pain and malaise. Cases of its transmission also have been reported via perinatal, sexual and transfusion transmission. ZIKV pathologies include meningo-encephalitis and myelitis in the central nervous system (CNS) and Guillain-Barré syndrome and acute transient polyneuritis in the peripheral nervous system (PNS). Drugs like azithromycin have been tested as inhibitors of ZIKV infection but no vaccines or treatments are currently available. Astrocytes are the most abundant cells in the CNS and among the first cells in CNS infected by ZIKV; (2) Methods: We previously used SOMAScan proteomics to study ZIKV-infected astrocytic cells. Here, we use mass spectrometric analyses to further explain dysregulations in the cellular expression profile of glioblastoma astrocytoma U251 cells. We also knocked down (KD) some of the U251 cellular proteins using siRNAs and observed the impact on ZIKV replication and infectivity; (3) Results & Conclusions: The top ZIKV dysregulated cellular networks were antimicrobial response, cell death, and energy production while top dysregulated functions were antigen presentation, viral replication and cytopathic impact. Th1 and interferon signaling pathways were among the top dysregulated canonical pathways. siRNA-mediated KD of HLA-A, IGFBP5, PSMA2 and HSPA5 increased ZIKV titers and protein synthesis, indicating they are ZIKV restriction factors. ZIKV infection also restored HLA-A expression in HLA-A KD cells by 48 h post-infection, suggesting interactions between this gene product and ZIKV.

## 1. Introduction

Zika virus (ZIKV) belongs to the family *Flaviviridae* and contains a 11 kb long positive single stranded (ss) RNA genome encoding 3 structural and 7 non-structural proteins surrounded by a nucleocapsid, made from viral capsid protein, enveloped in a host-derived lipid bilayer [1,2]. First isolated from non-human primates in 1947, ZIKV outbreaks date back to 2013 [2,3]. 2016 marked the peak number of cases in the US according to the CDC [4]. WHO reported 80 countries with cases of ZIKV transmission as of Feb 2022 [5]. ZIKV transmission is primarily by *Aedes aegyptii* mosquitoes [6]. ZIKV also can be transmitted in utero or by sexual and transfusion methods [5,7,8,9,10]. However, these epidemiological results are far from accurate because 80% of infections are asymptomatic or non-specific, lack of sufficient routine surveillance, and cross-reactivity with other flaviviruses like Dengue virus (DENV) [4,5,7]. Some of the symptoms associated with ZIKV infection include acute fever, conjunctivitis, muscle and joint pain, headache and arthralgia [11]. ZIKV pathology includes a multitude of neurological conditions such as Guillain-Barré Syndrome (GBS) and chronic inflammatory demyelinating polyneuropathies (CIDP) of the peripheral nervous system (PNS) and myelitis, acute disseminated encephalomyelitis (ADEM) and meningoencephalitis of the central nervous system (CNS) [12,13,14,15,16,17]. Other pathologies include acute onset chronic inflammatory demyelinating polyneuropathies, demyelination, axonal injury and other nervous system related conditions [12,18,19]. Some of the symptoms associated with these conditions include mild weakness to severe acute flaccid paralysis in the case of GBS [20]. Inflammation of the spinal cord and sensory-autonomic impairment has been associated with ZIKV cases of myelitis [21,22,23]. Inflammation of the brain, meninges, headaches, fever, seizures and aphasia in the case of meningoencephalitis, and a chronic progressive symmetric weakness in proximal and distal muscles in the case of CIDP have also been associated with ZIKV infections [18,22,24,25]. ZIKV infects astrocytes, oligodendrocytes, microglia, and neural progenitor cells, of which astrocytes form the largest cell population [26,27,28]. In utero studies in ZIKV-infected newborns associate intracranial calcifications, microcephaly, and cortical thinning and blindness with ZIKV infection [29,30,31]. Moreover, ZIKV also damages the blood–brain barrier (BBB) integrity via the increased production of inflammatory molecules leading towards viral persistence and replication in the CNS [32,33,34]. Choroid plexus and meninges are also prone to ZIKV infection which provides an additional pathway for ZIKV into the brain parenchyma [35,36]. Drugs like azithromycin have been tested as inhibitors of ZIKV infection but no vaccines or treatments are currently available [37].

Astrocytes perform numerous functions. They behave as living scaffolds for neurons, promoting neurogeneration [38]. Astrogliosis disruption can damage glial scar formation and maintenance of BBB processes [39,40]. Astrocytes also homeostatically control glutamate, lactate and Ca^++^ and K^+^ ions as part of their regulation of energy metabolism, oxidative stress and synaptic control [33,41,42]. This homeostatic control is crucial for normal muscle movement and behavioral control and if this homeostatic control of these ions does not exist in the CNS, it can result in inflammation, neurotoxicity and Huntington disease related implications [39,40,43,44]. Astrocytes are among the first cells to be infected by ZIKV.

Numerous mass spectrometry studies have been conducted in numerous cell types to examine host protein modifications after ZIKV infection. A label-free approach using LC-MS/MS in microcephalic fetuses found patterns of dysregulations in DNA damage repair response and mRNA translation to be linked with ZIKV infection [45]. Another TMT 10-plex study on placental tissues associated ZIKV infection with placental integrity compromise [46]. Mass spectroscopy on 124 serum samples identified fibrinogen alpha platelet factor 4 variant 1 (PF4V1) as a key difference in ZIKV and DENV infections in 62 patients [47]. ZIKV protein interaction mapping in HEK293T cells highlighted potential novel antiviral drug avenues against ZIKV infection [48]. LC-MS/MS studies in human fetal neural progenitor cells and on astrocytic cells, respectively, identified dysregulations in cell proliferation, differentiation and migration and neural cell adhesion molecule (NCAM1) as a receptor for ZIKV infection [26,49,50]. iPSC-derived astrocytic cells were also studied and linked ZIKV infection to DNA breakage and reactive oxygen species imbalance in the cells [51,52]. RNA approaches to understanding ZIKV infection have also yielded promising results. An RNA-seq study with mouse primary astrocytes revealed pathways related to neuron development, tight junction formation and astrocyte projection to be impacted by ZIKV infection [53]. We have previously used SOMAScan, an aptamer-based proteomic tool, to delineate astrocytic responses to ZIKV infection [54]. The SOMAScan platform uses slow off-rate modified aptamers (SOMAmers) that bind to proteins in their native state and are measured using DNA microarray chips [55]. Here, we used TMT 6-plex, coupled with LC-MS/MS to identify approximately 8000 astrocytic proteins. We also knocked down several of the identified dysregulated proteins and found that KD of several, including major histocompatibility complex class IA (HLA-A), heat shock protein family A member 5 (HSPA5), insulin like growth factor binding protein 5 (IGFBP5) and proteosome 20S subunit alpha 2 (PSMA2) led to increased ZIKV NS1 protein production and ZIKV titers, which suggests each of these proteins normally restrict ZIKV growth in astrocytic cells. 

## 2. Materials and Methods

### 2.1. Cells 

Human glioblastoma astrocytoma (U251) cells [U251 MG (previously known as U-373 MG; European Collection of Authenticated Cell Cultures—ECACC 09063001, Sweden)] were grown in Dulbecco’s modified Eagle’s/Nutrient Mixture F-12 (DMEM/F-12) media completed with 10% fetal bovine serum (FBS), 2 mM L-glutamine, 1× non-essential amino acids and 1× sodium pyruvate at 37 °C in 5% CO_2_. Cells were trypsinized every 2–3 days for passage. Viral titers were determined using plaque assays in Vero cells, as described [54].

### 2.2. Viral Infections 

Zika virus (ZIKV), Asian strain, was a gift from Dr. David Safronetz, Chief of Special Pathogens, National Microbiology Laboratory, Public Health Agency of Canada, Winnipeg, MB, Canada. ZIKV was grown in Vero cells in 1× DMEM containing 2.5% FBS, 2 mM L-glutamine, 2× gentamicin, 2× Amphotericin B, 1× non-essential amino acids and 1× sodium pyruvate at 37 °C in 5% CO_2_. Stock virus was prepared by low multiplicity infection (MOI = 0.001) and viral supernatants harvested at 3–5 days post-infection, supplemented with FBS to 20% and aliquots frozen at −80 °C until used. For experimental infection, 60–70% confluent U251 cells in 10 cm dishes or in 6-well plates were infected at an MOI of 3 to ensure >95% cells were infected at time 0. Virus was adsorbed at 37 °C in 5% CO_2_ for 2 h with frequent rocking. After 2 h, cells were overlayed with 1× DMEM/F12 media supplemented with 2.5% FBS (with 2 mM L-glutamine, non-essential amino acids and sodium pyruvate, 2× gentamicin and 2× amphotericin B). Parallel mock-infected samples also were prepared. Cells were scraped and the supernatants harvested at 12, 24 and 48 h post infection (hpi) and stored at −80 °C.

### 2.3. Plaque Assays

Plaque assays were performed in Vero cells grown in 12-well plates to 80–90% confluency. Dilutions of viral samples were prepared from 10^−1^ to 10^−6^ in gel saline (137 mM NaCl, 0.2 mM CaCl_2_, 0.8 mM MgCl_2_, 19 mM HBO_3_, 0.1 mM Na_2_B4O_7_, 0.3% (wt/vol) gelatin) and duplicate wells were infected with each dilution. After a 2 h adsorption of 100 µL of each dilution at 37 °C in 5% CO_2_, a 1:1 ratio of 1.2% Type I agarose (Difco Laboratories, Detroit, MI, USA) and (2× completed Medium 199–Medium 199 with 6% FBS, 4 mM L-glutamine, 10 µg of gentamicin sulfate per mL and 3 µg of amphotericin B per mL) was overlayed onto the cells. Cells were incubated at 37 °C in 5% CO_2_ for 4 days. Plates were stained with 0.04% neutral red in 1% agar in PBS. The plaques were counted 18–21 h post staining.

### 2.4. Cell Lysis and Quantification of Proteins

Cell pellets from each of 18 samples (3 replicates each of infected samples at 12, 24, and 48 hpi, and time-matched non-infected mock samples) were lysed using MPER^®^ (Pierce; Rockford, IL, USA) solution supplemented with 1× HALT^®^ protease inhibitor (Pierce; Rockford, IL, USA), centrifuged at 14,000× *g* for 12 min at 4 °C and the lysate separated from the cellular debris. BCA protein assay (Pierce; Rockford, IL, USA) was used to quantify the amount of protein in each sample using bovine serum albumin (BSA) as a standard.

### 2.5. Mass Spectrometry

All protein samples were processed using the single-pot solid-phase-enhanced sample preparation (SP3) protocol to remove any detergent and salts from the lysates [43]. Lysates were first reduced by DTT to break tertiary structures including disulfide linkages, and then alkylated using iodoacetamide to prevent re-oxidation. After reduction and alkylation, the proteins were purified using the SP3 carboxylate modified Sera-Mag^TM^ beads. The beads were washed 2× with 70% ethanol then by 100% ACN. After the washing steps, trypsin was added at an enzyme to protein ratio of 1:25 and the proteins were digested into peptides, eluted and labelled with the TMT 6-plex system. Labelled samples were analyzed by liquid chromatography/tandem mass spectrometry (LC -MS/MS). 

### 2.6. siRNA-Mediated Knock Down

For initial screens, 20 μM stock solutions of lyophilized siRNA were prepared by dissolving them in 1× siRNA buffer. 5000 U251 cells in each well of a 96-well plate were treated with 80 nM of each of various SMARTPool On-Target plus siRNA targeting a variety of cellular genes. Scrambled control non-silencing siRNA were used for the negative control treatment. Dharmafect/OptiMEM mixture was used for the transfection. After siRNA treatment, cells were incubated at 37 °C for 48 h, after which their cell viability was measured by WST-1 assay (described below). At 48 h post siRNA treatment (hpt), U251 cells were infected with ZIKV at an MOI of 3 (described above) and the supernatants were collected for viral titer measurements using plaque assays. All the experiments were performed in triplicates.

To knockdown specific genes, 20 µM stock solutions of lyophilized siRNA were prepared by dissolving them in 1× siRNA buffer. Knockdown of HLA-A, HSPA5, IGFBP5 and PSMA2 was performed using the SMART-Pool siRNAs for each at 25 nM. Scrambled siRNA was used as negative control. U251 cells were plated in 6-well plates, grown to 40% confluency and siRNA, solubilized in OptiMEM/Dharmfect, were added. Cells were treated with siRNAs for 48 h. In some cases, a second treatment was performed 24 h after the first siRNA treatment. Afterwards, ZIKV infections were done at an MOI of 3 and supernatants and cell pellets were collected for viral plaque assay and viral protein synthesis at 48 hpi. The experiments were performed in triplicates.

### 2.7. WST-1 Assay

Cell viability was determined using the WST-1 assay in 96-well plate format. Cells in each well were treated with 8 µL of WST-1 reagent after 48–72 h of siRNA treatment and incubated at 37 °C for 1.5 h. Absorbances at 440 nm and 610 nm were recorded using a plate reader and 610 nm absorbance values of infected samples were subtracted from their corresponding 440 nm absorbance values before being normalized to their respective mock samples. An average of 4–6 replicates per condition was calculated to determine cell viability values.

### 2.8. Western Blotting

10–20 μg of protein lysates were combined with 10% 2-mercaptoethanol, heated for 5 min at 95 °C, and resolved in 12% sodium dodecyl sulfate polyacrylamide electrophoresis (SDS-PAGE) gels at 100–120 V until the loading dye reached the bottom of the gel. Resolved proteins were transferred to PVDF membranes (Immobilon-P polyvinylidene difluoride membrane (Millipore), Sigma-Aldrich Canada Co., Oakville, ON, Canada). The PVDF membranes were blocked with 5% skim milk (wt/vol in 1× TBST) for 1 h at room temperature or overnight at 4 °C. Primary antibodies were mouse anti-ZIKV NS1 (BioFront Technologies # BF-1225-06, Tallahassee, FL, USA), mouse anti-beta actin (Cell Signaling # 8H10D10, Danvers, MA, USA), rabbit anti-HLA-A (GeneTex # GTX114080 N1C2, Irvine, CA, USA), rabbit anti-IGFBP5 (Cell Signaling #3488, Danvers, MA, USA), mouse anti-HSPA5 (EMD Millipore Corp. MABC6750 clone 4E3, Oakville, ON, Canada), rabbit anti-PSMA2 (Cell Signaling 2455S, Danvers, MA, USA) and rabbit anti-GAPDH antibodies. Secondary antibodies used were goat HRP-conjugated anti-rabbit (Cell Signaling # 7074, Danvers, MA, USA) and goat HRP-conjugated anti-mouse (Cell Signaling # 7076, Danvers, MA, USA). Blots were imaged using ECL Western blotting peroxidase substrate for chemiluminescence. ImageJ was used to quantify the Western bands, and each was then normalized to their respective actin or GAPDH controls. 

### 2.9. Statistical and Bioinformatic Analyses

Numerous peptide sequences were identified by mass spectrometric analyses and proteins were identified and quantified from at least 2 different non-redundant peptides in its sequence. This resulted in expectation values and calculated false discovery values of 0.1%, using the xTandem (https://www.thegpm.org, accessed on 28 October 2022) peptide identification software. Protein quantity fold changes between infected and each time-matched mock samples were converted to log_2_ values and significance determined by Students *t*-test and by Z-score analyses as described [54]. 

## 3. Results

### 3.1. The Numbers of Significantly Dysregulated U251 Proteins Increase with Time in ZIKV-Infected Cells

Table 1 shows the numbers of statistically significant proteins in ZIKV-infected U251 cells at 12, 24 and 48 hpi at different fold-change (F.C.) cut off values. The number of statistically significant proteins increases over time and decreases as the fold-change values are made more stringent. For example, F.C. cut off values of >1.333 and <0.750 identified 33 dysregulated proteins (24 over-expressed and 9 under-expressed) at 12 hpi, 110 (89 over-expressed and 21 under-expressed) at 24 hpi and 200 (135 over-expressed and 65 under-expressed) at 48 hpi (Table 1). However, more stringent F.C. > 1.500 and <0.667 resulted in 19 dysregulated proteins at 12 hpi, 73 at 24 hpi and 106 at 48 hpi (Table 1). Only 50 U251 cellular proteins were significantly over-expressed or under-expressed at a F.C. cut-off of +/- 2.5 (Table 2). 33 proteins were under-expressed while 17 were over-expressed in ZIKV-infected U251 cells compared to their time-matched mock-infected U251 samples. Examples of ZIKV-mediated under-expressed proteins include STC1 (−14.47-fold), IGFBP5 (−6.90-fold), and MDK (−5.02-fold) at 48 hpi (Table 2). Examples of ZIKV-mediated over-expressed proteins include OAS2 (3.87-fold), HLA-B (4.20-fold), and CXCL11 (8.61-fold) at 48 hpi (Table 2). The most over-expressed protein was CXCL11 (8.61-fold) and the most under-expressed protein was STC1 (−14.47-fold) (Table 2). We applied F.C. cut off values of >1.3 and <0.750 to provide sufficient stringency to the data while keeping the number of dysregulated targets high enough for subsequent bioinformatic analyses.

### 3.2. Numerous U251 Cellular Networks and Proteins Are Dysregulated by ZIKV at 48 hpi

**Figure 1:**
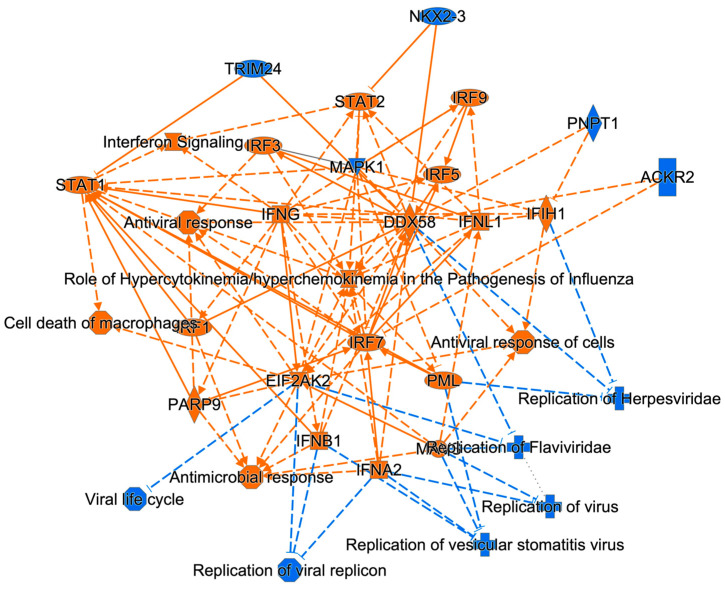
Ingenuity Pathway Analysis (IPA)-generated graphical summary of ZIKV-dysregulated patterns of U251 proteins and cellular functions based on the list of statistically significantly dysregulated proteins in U251 cells at 48 hours post ZIKV infection (hpi). The organization and functional linkages result from IPA default settings and using protein fold-change cut-off values of >1.33 and <−1.33 when comparing ZIKV-infected protein abundances to time-matched non-infected protein abundances. Orange: predicted activation (as generated by IPA) Blue: predicted inhibition. Alpha value is 0.05. Statistical significance was determined using 2-tailed Students *t*-test and Z-score analyses.

The largest number of significantly dysregulated proteins and cellular networks were dysregulated by ZIKV at 48 hpi and these pertain to viral replication, antiviral responses, interferon signaling, and antimicrobial responses (Figure 1). Some of the proteins involved in each of these top dysregulated cellular networks include IFNG, IFNIL1, IRF5, PARP9 and STAT2 (Figure 1).

**Figure 2:**
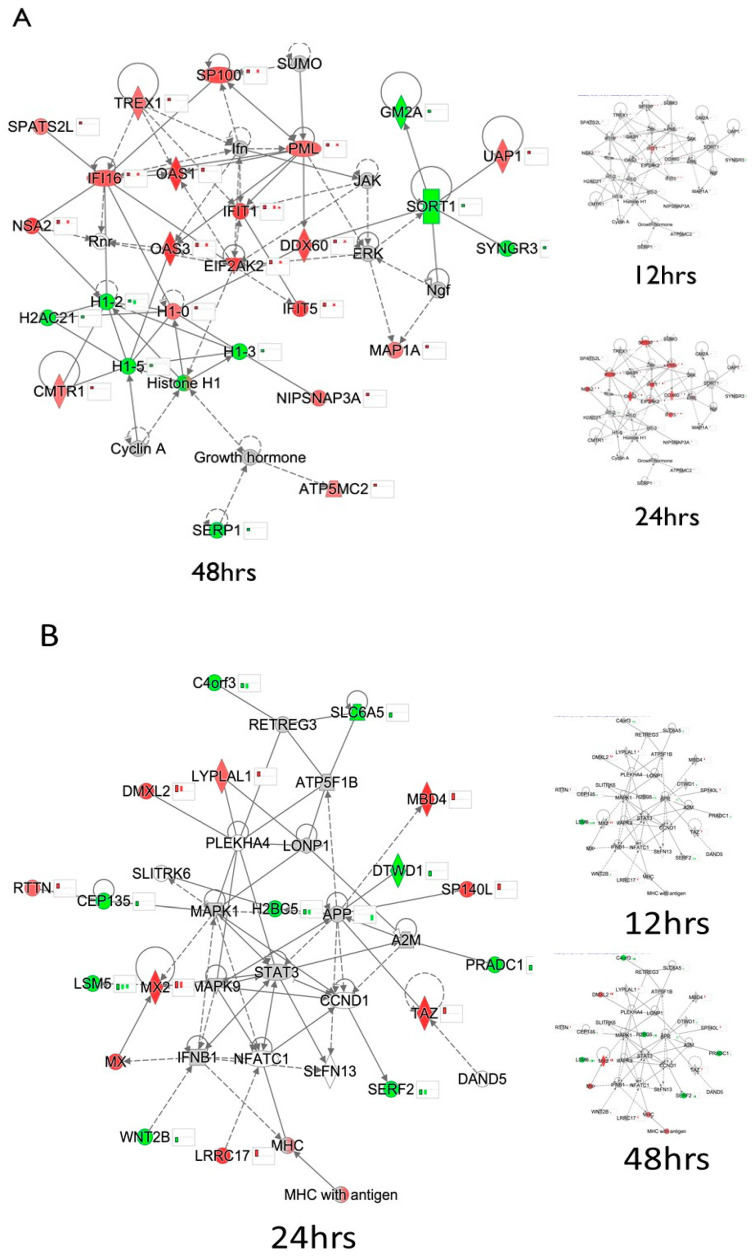
Top dysregulated cellular networks in ZIKV-infected U251 cells at 24 and 48 hpi, respectively, based on the list of statistically significantly dysregulated proteins (**A**)**,** Top dysregulated cellular network in ZIKV-infected U251 cells at 48 hpi is Antimicrobial Response, cell signaling and infectious diseases. Overlaid 12 and 24 hpi data shown in smaller diagrams. (**B**), Top dysregulated cellular network in ZIKV-infected U251 cells at 24 hpi is Cell Death and survival, connective tissue development and function and energy production network. Overlaid 12 and 48 hpi data shown in smaller diagrams. Green represents U251 proteins that were significantly under-expressed compared to their time-matched mock-infected values while red represents proteins that were significantly over-expressed compared to their time-matched mock-infected samples. 2-tailed Students *t*-test and Z-score analyses were used to determine the statistical significance.

The top dysregulated cellular network at 24 hpi was cell death and survival, connective tissue development and function and energy production, while the top-most dysregulated network at 48 hpi was antimicrobial response, cell signaling and infectious diseases (Figure 2A,B). Data from other time points were overlayed on the networks to show the time progression of dysregulation. Most proteins in the overlayed data had either reduced dysregulation by ZIKV or non-significant dysregulation by ZIKV. Two examples are OAS3 at 48 h and MBD4 at 24 h, both of which show differential expression at other timepoints, providing more understanding of the progressive effect of ZIKV on their expressions. Other examples include OAS1, IFI16 and MAP1A which were over-expressed at 48 hpi and SORT1, GM2A and SERP1 which were under-expressed at 48 hpi (Figure 2A). At 24 hpi, some of the over-expressed proteins include MBD4, DMXL2, MX2 and LRRC17 while some of the under-expressed include SERF2, WNT2B and LSM6 (Figure 2B). This shows ways the virus hijacks cellular machinery that consequently results in the dysregulation of important astrocytic functions.

### 3.3. Numerous Cellular Functions and Canonical Pathways Are Activated or Inhibited at 48 hpi

**Figure 3:**
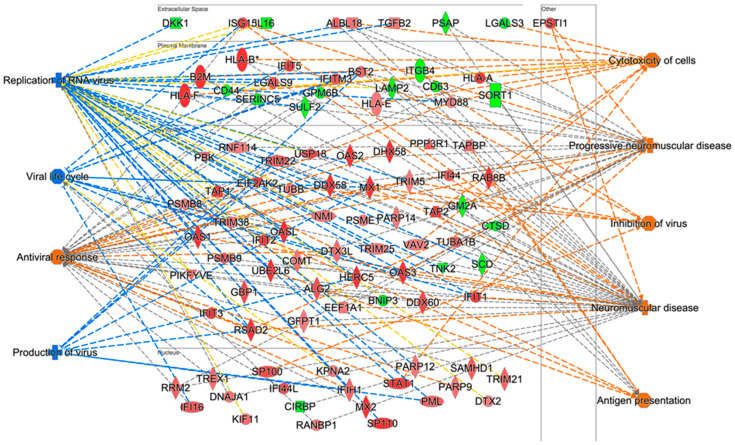
Predicted dysregulation of host cell diseases and functions at 48 hpi. Cut off *p*-value is 0.05. Z-score threshold is +/− 1.96σ. Green represents U251 proteins that were significantly under-expressed by ZIKV infection compared to their time-matched mock-infected samples, while red represents proteins that were significantly over-expressed by infection compared to their time-matched mock-infected samples. 2-tailed Students *t*-test and Z-score analyses were used to determine the statistical significance. Orange represents predicted activation of the cellular function and disease, and blue represents prediction inhibition as generated by IPA software based on the statistically significant data from the comparison of ZIKV-infected U251 cells to time-matched mock-infected U251 cells at 48 hpi.

**Figure 4:**
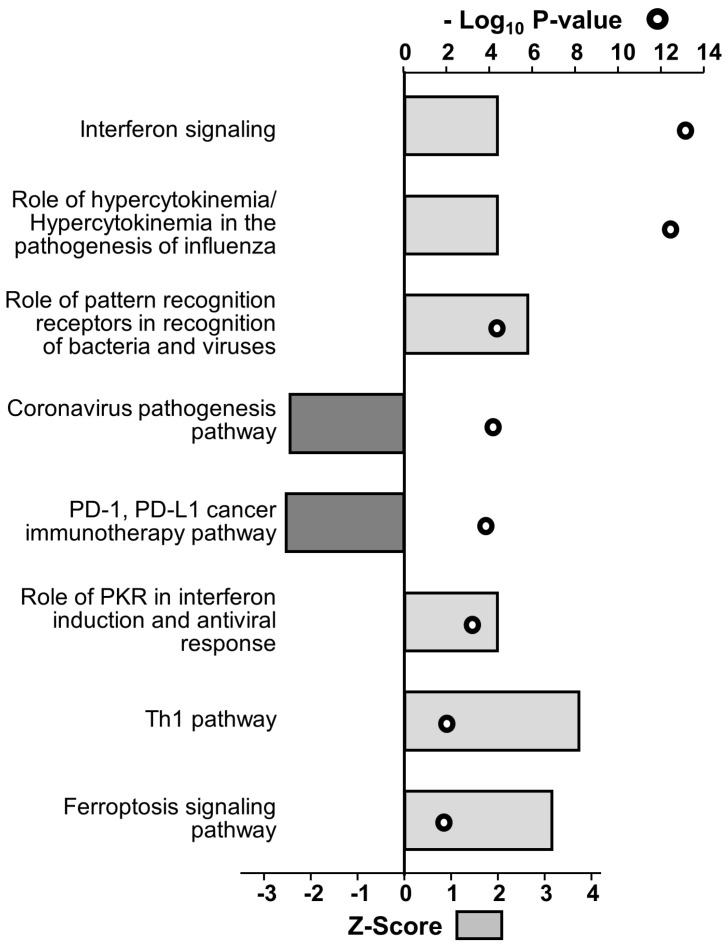
ZIKV—induced top dysregulated canonical pathways based on the expression profile of U251 cells at 48 hpi, as generated by IPA software. Light grey bars are predicted activation and dark grey bars are predicted inhibition based on the list of significantly dysregulated proteins at 48 h post ZIKV infection in U251 cells found by mass spectrometry. 2 tailed Students T—test was used to determine statistical significance (top axis), and degree of predicted activation/inhibition was determined by Z-score (bottom axis). For each of them, the cut off *p*-value is 0.05 and the cut off Z-score value is +/− 1.96σ.

Since the largest number of significantly dysregulated proteins were at 48 hpi, top dysregulated cellular functions are depicted in Figure 3. Predicted activation was identified for cellular functions such as cytotoxicity of cells, progressive neuromuscular disease, antigen presentation and neuromuscular disease, while predicted inhibition was identified for cellular functions namely replication of virus and viral life cycle (Figure 3). For example, over-expressed proteins such as HLA-A, HLA-B and HLA-E and under-expressed proteins such as LGALS3 and SORT1 contribute to cytotoxicity of cells (Figure 3). Some of the U251 activated canonical pathways at 48 hpi include interferon signaling, role of pattern recognition receptors in recognition of bacteria and viruses, role of PKR in interferon induction and antiviral response and Th1 pathway, while some of the IPA predicted inhibited canonical pathways include PD-1, PD-L1 cancer immunotherapy pathway and coronavirus pathogenesis pathway (Figure 4).

### 3.4. Knocking Down U251 Genes Affects ZIKV Growth 

**Figure 5:**
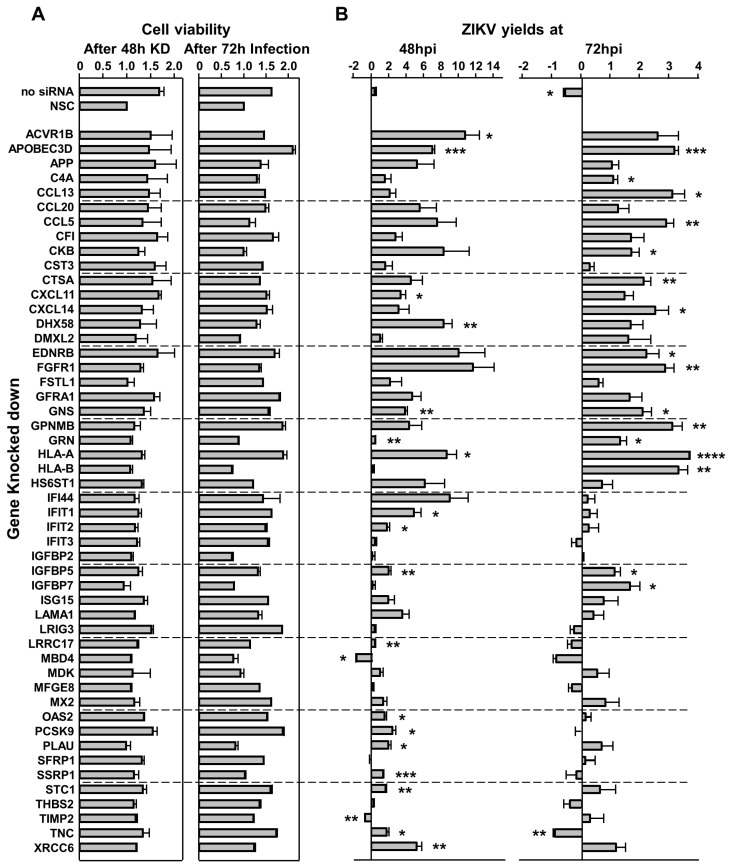
Effect of U251 gene knockdown (KD) on cell viability (**A**) and ZIKV yields (**B**). Cells were treated with 80 nM of the indicated siRNA (left). (**A**), Cell viabilities determined by WST − 1 assay at 48 h post − siRNA treatment without ZIKV infection (left) and after an additional 72 h after ZIKV infection (right). (**B**)**,** ZIKV yields from indicated KD cells at 48 (left) and 72 hpi (right). Cell viabilities and ZIKV yields are compared to those in U251 cells treated with non-silencing scrambled control (NSC). Error bars show standard deviation. * *p*-value < 0.05, ** *p*-value < 0.01, *** *p*-value < 0.001, **** *p*-value < 0.0001.

To ascertain the effects of disrupting proteins that were either over-expressed or under-expressed by ZIKV infection, we selected 50 cellular genes and knocked them down by siRNA treatment. Cell viability of non-infected cells after KD was >90% (Figure 5A). Cell viability of most infected KD cells also were not significantly reduced, except for IGFBP2, HLA-B, IGFBP7 and MBD4, which resulted in approximately 40% reduction in viability (Figure 5A). KD of most proteins resulted in a 2-fold to 10-fold increase in viral titers while others decreased the viral titers by 1.5 to 2-fold (Figure 5B). For example, APOBEC3D, GNS, GRN, HLA-A and IGFBP5 siRNA treatments increased ZIKV titers at both timepoints (Figure 5B). APOBEC3D siRNA caused 7- and 3-fold increase, HLA-A siRNA caused 8- and 4-fold increase, and IGFBP5 caused 3- and 1.5-fold increase at 48 hpi and 72 hpi compared to NSC-treated U251 cells (Figure 5B). Other siRNAs, such as MBD4, decreased viral titers at either time point by roughly 2-fold. TIMP2 KD reduced viral titers by roughly 1.4-fold.

### 3.5. HSPA5, PSMA2, IGFBP5 and HLA-A KD Result in Increased ZIKV NS1 Expression and Titers

**Figure 6:**
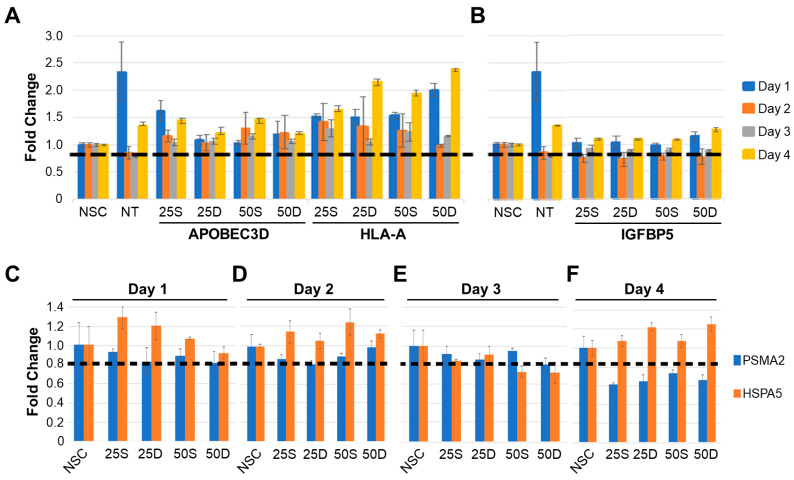
Optimization of siRNA treatments, number of siRNA treatments and time kept in the siRNA treatment in U251 cells. The dashed bar represents 80% of cell viability compared to the non-silencing scrambled control (NSC). Cells were plated at 30–40% confluency to ensure that by day 4, the wells were close to 95% confluent and overcrowding was not occurring. (**A**), Cell viability results for U251 cells treated with APOBEC3D and HLA-A siRNA for 1–4 days. Error bars represent standard deviations. (**B**), Cell viability results for U251 cells treated with IGFBP5 siRNA for 1–4 days. Error bars represent standard deviations. UT represents cells that have not been treated with any siRNA at all, while NSC represents cells that have been treated with non-silencing control siRNA. UT condition is only included to see if any changes in cell viability occur due to presence of siRNA itself. Cell viability results for U251 cells treated with PSMA2 or HSPA5 siRNA treatment for (**C**), 1 day; (**D**), 2 days; (**E**), 3 days; and (**F**), 4 days. Blue bars indicate PSMA2 siRNA treatment cell viabilities; orange bars indicate HSPA5 siRNA treatment cell viabilities. S: single siRNA treatment; D: double siRNA treatment. Error bars represent standard deviations.

KDs of several genes were initially optimized. Cell viabilities did not decrease by day 4 post-treatment after 25 nM and 50 nM siRNA treatment with APOBEC3D and HLA-A siRNAs (Figure 6). HLA-A KD increased cell viability by about 140% (to 240%) compared to non-treated control (NTC) by day 4 (Figure 6A). Both 25 nM and 50 nM IGFBP5 siRNA treatments decreased cell viability to about 85% by day 2 and increased it back to 110% and 130% by day 4 (Figure 6B). Cell viability results for PSMA2 and HSPA5 siRNA treatments are shown in Figure 6D–F. PSMA2 siRNA treatment decreased the cell viability to 0.6-fold of non-treated by day 4, while HSPA5 siRNA treatment decreased it to 0.7 fold by day 3 and increased it to 1.2 fold by day 4 (Figure 6C,D). 

**Figure 7:**
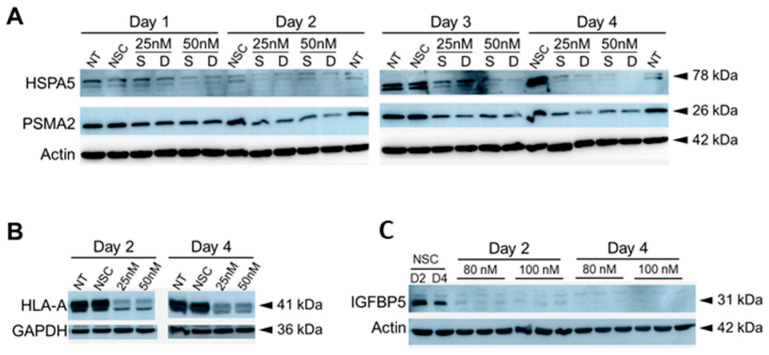
Western blot verification of siRNA-mediated knockdown after (**A**), HSPA5 or PSMA2; (**B**), HLA-A; or (**C**), IGFBP5 knockdown at indicated days post siRNA treatment in U251 cells. Various genes were knocked down with 25, 50, 80, or 100 nM, single (S) or double (D) siRNA treatment. UT: cells not treated with any siRNA; NSC:, cells treated with 100 nM scrambled non-silencing control siRNA. Actin or GAPDH were used as loading controls.

A single 50 nM treatment with HSPA5 and PSMA2 siRNA was more effective at knocking down HSPA5 and PSMA2 protein expression in U251 cells 2 and 4 days post-treatment, while 25 nM single treatment of PSMA2 and HSPA5 was more effective in knocking down by day 4 (Figure 7A). For HLA-A, single treatment of 50 nM siRNA successfully knocked down HLA-A expression by day 2 and day 4 post siRNA treatment (Figure 7B). Both 80 nM and 100 nM concentrations of IGFBP5 siRNA knocked down the protein expression by days 2 and 4 post single treatment successfully (Figure 7C).

**Figure 8:**
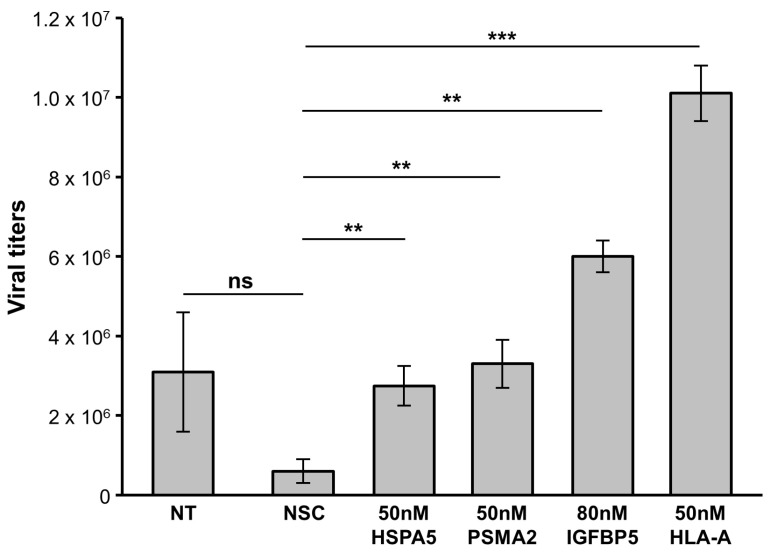
Effect of indicated siRNA treatment on ZIKV titers in U251 cells at 48 hpi after indicated genes and proteins knocked down. Error bars represent standard deviation. 2-tailed Students *t*-test was used to determine statistical significance and *p*-values. * *p*-value < 0.05; ** *p*-value < 0.01; *** *p*-value < 0.001; ns: not significant; NT; not treated with siRNA; NSC: scrambled non-silencing control.

**Figure 9:**
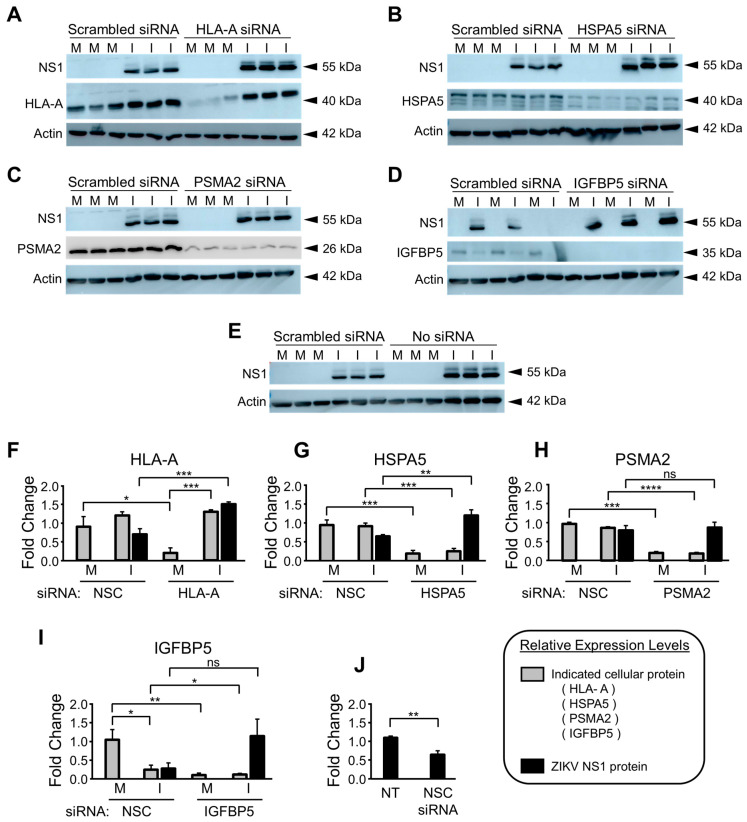
Effect of siRNA treatment on ZIKV NS1 expression in U251 cells at 48 hpi (**A**), NS1 and HLA-A protein expression in scrambled non-silencing siRNA vs. HLA-A siRNA-treated mock (M) and ZIKV-infected (I) U251 cells. (**B**), NS1 and HSPA5 protein expression in scrambled siRNA and HSPA5 siRNA-treated mock vs. ZIKV-infected U251 cells. (**C**), NS1 and PSMA2 protein expression in scrambled siRNA and PSMA2 siRNA-treated mock vs. ZIKV-infected U251 cells. (**D**), NS1 and IGFBP5 protein expression in scrambled siRNA and IGFBP5 siRNA-treated mock vs. ZIKV-infected U251 cells. (**E**), NS1 protein expression in scrambled siRNA and non-treated (no siRNA) mock vs. ZIKV-infected U251 cells. (**F**–**J**) show the protein quantifications of each of the bands for siRNA targeted proteins and ZIKV NS1 protein shown in (**A**–**E**), respectively. Errors bars represent standard deviation and 2-tailed Students *t*-test was used for statistical significance and to obtain *p*-values. * *p*-value < 0.05, ** *p*-value < 0.01, *** *p*-value < 0.001, **** *p*-value < 0.0001. M: mock; I: infected.

50 nM concentrations of PSMA2, HSPA5 and HLA-A siRNAs increased ZIKV titers in U251 cells by 48 hpi, compared to those in non-silencing control siRNA-treated cells (Figure 8). PSMA2 KD resulted in a 3-fold increase, HSPA5 KD in a 2-fold increase, HLA-A KD in a 10-fold increase, and IGFBP5 KD in a 6-fold increase in ZIKV titers (Figure 8). ZIKV NS1 expression was increased in HLA-A, HSPA5 and IGFBP5 KD cells by 48 hpi but not in PSMA2 KD cells (Figure 9A–D).

More NS1 was expressed in non-treated U251 cells compared to scrambled siRNA-treated U251 cells (Figure 9E). ZIKV infection restored HLA-A expression in HLA-A KD U251 cells by 48 hpi (Figure 9A). HSPA5 KD, PSMA2 KD and IGFBP5 KD remained stable throughout the course of infection (Figure 9B–D). Western blot quantification of the siRNA KD proteins and ZIKV NS1 in each condition is shown in Figure 9F–J, respectively. HLA-A KD treatment decreased HLA-A expression to 0.2-fold that of the non-silencing siRNA-treated U251 cells (Figure 9F). ZIKV restored the expression of HLA-A in HLA-A KD conditions by 2.5 fold by 48 hpi (Figure 9F). ZIKV NS1 expression increased 2-fold in the presence of the HLA-A KD by 48 hpi (Figure 9F). HSPA5 KD decreased HSPA5 expression by 5-fold and the KD remained stable in the presence of ZIKV infection (Figure 9G). HSPA5 KD increased ZIKV NS1 expression by 1.8-fold (Figure 9G). PSMA2 KD decreased PSMA2 expression 5-fold and it remained stable in ZIKV infected conditions by 48 hpi (Figure 9H). PSMA2 KD did not impact ZIKV NS1 expression in U251 cells by 48 hpi (Figure 9H). IGFBP5 siRNA decreased IGFBP5 expression 8-fold in U251 cells and it remained stable in ZIKV-infected conditions by 48 hpi (Figure 9I). ZIKV infection alone was also able to reduce IGFBP5 expression in U251 cells at 48 hpi (Figure 9I). IGFBP5 siRNA treatment increased ZIKV NS1 expression 2-fold compared to that of non-silencing control siRNA treatment, despite not being statistically significant (Figure 9I). Finally, ZIKV NS1 expression was 0.6-fold in the non-silencing siRNA treated U251 cells compared to that in non-treated U251 cells by 48 hpi (Figure 9J).

### 3.6. ZIKV Infection Causes Restoration of HLA-A Levels in HLA-A KD Cells

**Figure 10:**
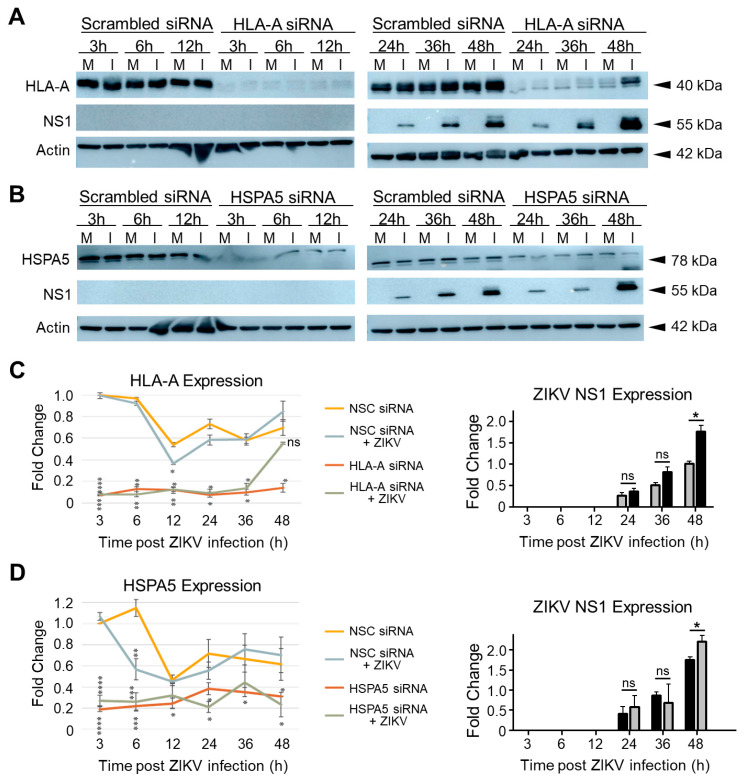
Expression of siRNA targeted proteins and ZIKV NS1 in ZIKV-infected siRNA-treated KD U251 cells at multiple timepoints. (**A**), ZIKV NS1 and HLA-A protein expression in scrambled siRNA vs. 50 nM HLA-A siRNA-treated, mock vs. infected U251 cells at 3, 6, 12, 24, 36 and 48 hpi. (**B**), ZIKV NS1 and HSPA5 protein expression in scrambled siRNA vs. 50 nM HSPA5 siRNA-treated mock (M) and infected (I) U251 cells at 3, 6, 12, 24, 36 and 48 hpi. (**C**,**D**) show quantification of bands for siRNA targeted proteins and ZIKV NS1 in (**A**,**B**), respectively. All error bars are standard deviations and 2-tailed Students t-test was used to measure statistical significance and calculate *p*-values. * *p*-value < 0.05, ** *p*-value < 0.01, *** *p*-value < 0.001, **** *p*-value < 0.0001. ns means not significant.

HLA-A KD increased ZIKV NS1 expression over time with 24 hpi being the first time point when any NS1 expression was detected (Figure 10A). HLA-A expression was partially restored in HLA-A KD U251 cells after 24 hpi and the restoration of HLA-A expression increased by 48 hpi (Figure 10A). HSPA5 KD increased ZIKV NS1 expression over time with the first expression being observed at 24 hpi (Figure 10B). HSPA5 KD remained unaffected by ZIKV infection (Figure 10B). Western blot quantification of siRNA-mediated KD proteins and ZIKV NS1 in each of the KD conditions at 3, 6, 12, 24, 36, and 48 hpi in comparison to non-silencing control are shown in Figure 10C,D, respectively. HLA-A siRNA treatment decreased HLA-A expression by 9-fold compared to that in scrambled siRNA-treated U251 cells by 3 hpi, irrespective of the presence of ZIKV infection (Figure 10C). Over time, the HLA-A-siRNA mediated KD remained stable (Figure 10C). ZIKV restored the expression of HLA-A and increased it by 6-fold in comparison to the HLA-A KD mock-infected U251 cells by 48 hpi (Figure 10C). HLA-A KD increased ZIKV NS1 expression more than scrambled siRNA (Figure 10C). HLA-A KD in U251 cells increased ZIKV NS1 expression by 1.8-fold compared to that in scrambled siRNA-treated U251 cells by 48 hpi (Figure 10C). Earlier time points also showed increased ZIKV NS1 expression in HLA-A KD U251 cells than in time-matched scrambled siRNA-treated ZIKV-infected U251 cells despite not being statistically significant (Figure 10C). HSPA5 KD decreased HSPA5 expression by 8-fold compared to the scrambled siRNA treatment by 3 hpi, irrespective of the presence of ZIKV infection (Figure 10D). Over time, HSPA5 KD remained stable in U251 cells although the levels of HSPA5 in scrambled siRNA-treated U251 cells also slightly decreased over time (Figure 10D). HSPA5 KD in U251 cells was unaffected by ZIKV infection (Figure 10D). HSPA5 KD increased ZIKV NS1 expression in U251 cells more than the scrambled siRNA (Figure 10D). Western quantification showed HSPA5 KD increasing ZIKV NS1 expression by 1.5-fold than that in ZIKV-infected scrambled siRNA-treated U251 cells by 48 hpi (Figure 10D). Earlier time points showed higher ZIKV NS1 expression in HSPA5 KD U251 cells than in the time-matched scrambled siRNA-treated ZIKV-infected U251 cells despite not being statistically significant (Figure 10D).

## 4. Discussion

We used a mass spectrometry-based proteomic approach to understand ZIKV infection in U251 cells, followed by siRNA-mediated knockdown studies to delineate the importance of different host cellular proteins in ZIKV replication and protein synthesis. Most of the proteomic pathway analyses were performed using IPA software to generate top ZIKV dysregulated cellular networks, functions and canonical pathways and they helped explain some of the ZIKV-mediated molecular dysregulations that could potentially lead to broader clinical manifestations. The fold-change cut-off was chosen to be >1.333 and <0.750 to increase the stringency, while keeping the number of statistically significant proteins large enough for meaningful bioinformatic analyses (Table 1). For example, OAS3, which negatively regulates interferon/chemokine responsive genes and modulates innate response against Chikungunya infection [24,56,57], was over-expressed (Figure 2A), while SERP1, which regulates stress responses and interacts with viral NS4B protein to suppress replication in cases of DENV type 2 infections [58,59], was under-expressed (Figure 2A). Similarly, MBD4, which has a role in DNA methylation, repair and CNS development [41,60,61], and MX2, which is a restriction factor for other viruses including HIV, were over-expressed (Figure 2B), while WNT2B, which regulates cell growth, adult CNS development and differentiation and innate responses via the CTNNB1 signaling pathway [62,63,64,65,66], was under-expressed (Figure 2B).

Among the ZIKV dysregulated cellular functions at 48 h post ZIKV infection, which was the timepoint with the largest number of dysregulated proteins, MAPK1 was predicted to be activated and STAT1/2 inhibited (Figure 1). MAPK1 dysregulation induces chorio-retinal atrophy and optic nerve abnormalities in ZIKV infections and STAT1/2 is involved in antiviral responses; in addition, ZIKV NS5 protein-mediated STAT2 degradation modulates type I and III interferon responses [51,67,68,69]. Other molecules dysregulated by ZIKV among the top ZIKV dysregulated functions at 48 hpi included over-expressed proteins such as STAT1, IFIT2/3 and HLA-A and under-expressed proteins such as LAMP2 (Figure 3). Many of them have crucial roles in healthy cellular functions and are also involved in other viral infections. STAT1 mediates antiviral type I, II and III interferon responses, regulates ZIKV-mediated induction of cholesterol 25-hydroxylase and interacts with STAT2 for its 

ZIKV infection regulation via ZIKV NS2A interaction [68,70,71,72,73]. IFIT2/3 regulate apoptotic processes, stabilize IFIT1 and promote its binding to viral RNA for translation inhibition [65,74,75]. HLA-A has a role in WNV and DENV infections and disease severity in HIV and SARS-CoV2 infections [76,77,78,79,80]. LAMP2 regulates lysosome biogenesis and autophagosome maturation in other viral infections such as African swine fever virus (ASFV) by interacting with ASFV E248R and E199L proteins and DENV infections [54,81,82,83,84]. Therefore, their roles in ZIKV infection need to be further examined to help better understand ZIKV modulation of astrocytic functions to aid its infection and replication.

Finally, several ZIKV-dysregulated canonical pathways including the Th1 pathway, role of pattern recognition receptors and interferon signaling highlight the role of ZIKV in neuroinflammation and CNS immune modulation as astrocytes are known to be involved in CNS tissue repair, inflammation, NF-kB pathway and MAPK pathways (Figure 4) [85,86]. Moreover, cognitive functions, synaptic plasticity and DNA and RNA viral load control via Type I interferon receptor (IFNAR) signaling are among the other astrocytic functions potentially dysregulated by ZIKV [85,87,88]. We had previously used the SOMAScan platform and identified ZIKV-induced U251 proteomic dysregulations [54]. Here, we combined the results from both studies, identified 50 proteins dysregulated at least 2.5-fold, either upwards or downwards, and ascertained what effects KD of each of these genes would have (Figure 6). Proteins like APOBEC3D and HLA-A not only increased ZIKV titers upon being KD but also cell viability (Figure 5). From the list of siRNA targets, APOBEC3D, HLA-A and IGFBP5 siRNA KD were among the few that increased ZIKV titers while MBD4, TIMP and TNC KD decreased them (Figure 7). APOBEC3D is a cytidine deaminase and inhibits other viruses such as HIV-1 and human cytomegaloviruses [89,90,91]. Unfortunately, the APOBEC3D antibody we tested did not work, so it was not followed up with. HLA-A class I & II have roles in WNV and secondary DENV infections, and in HIV and SARS-CoV2 disease severity [76,77,78,79,80]. IGFBP5 interacts with heparan sulfate proteoglycans (HSGs) and cell-surface matrix glycoproteins, which are receptors for HIV tat, HSV 1&2 and DENV [92]. PSMA2 and HSPA5 were included because we have found they are also involved in viral replication. PSMA2 is crucial for 20S proteosome complex assembly, degradation of damaged proteins and is involved in influenza-mediated escape from viral clearance via inhibition of NRF2-regulated oxidative stress response while HSPA5 facilitates binding, entry and protein folding for many viruses including Ebola, BDV, MERS-CoV, SARS-CoV2, DENV E and HBV [93,94,95,96,97]. After determining the concentration of siRNA that resulted in a successful KD by 48 hpi, with at least 60% cell viability, U251 cells were KD using siRNAs targeting PSMA2, HSPA5, HLA-A or IGFBP5 (Figure 6 and Figure 7). This resulted in an increase in ZIKV titers, highlighting their role as potential restriction factors in U251 cells (Figure 8). HLA-A and IGFBP5 had contrasting results. ZIKV increased HLA-A expression and reduced IGFBP5 expression but KD of each increased ZIKV titers (Figure 8). This highlights their differing roles in ZIKV replication. IGFBP5, an insulin like growth factor binding protein, is an IGF signaling regulator and is crucial for cell proliferation, growth and survival. Therefore, ZIKV decreasing its expression modulates growth and leads towards increased cell death as we reported previously [54]. In addition, IGFBP5 is an activator of PI3K/AKT and MAPK pathways which in turn are utilized by other viruses such as Ebola virus for cell entry [98]. Hence, later upon its KD, it could increase ZIKV efficiency to establish infection and replicate faster in U251 cells. However, HLA-A is a major histocompatibility complex antigen, ubiquitously expressed in nearly all nucleated cells with its role in endogenous peptide presentation to CD8+ T cells. Thus, increase in HLA-A in the presence of ZIKV could be a host response to viral infection and KD of it circumvents this response allowing faster replication (Figure 8). This highlights how KD of genes, either up- or down-regulated by the virus, can still exert differing effects on viral titers depending on the function and thus further studies are warranted.

Interestingly, ZIKV also restored HLA-A expression over time in HLA-A KD cells, highlighting potential cross-talk between HLA-A and ZIKV proteins (Figure 9 and Figure 10). This is interesting because HLA-A expression in astrocytes is mostly in cells confined to CNS lesions [99,100]. Moreover, HLA-A also has roles in antigen presenting cells (APC). Therefore, it will be interesting to look at how HLA-A directs ZIKV proteins, if at all, to the surface for antigen presentation [101,102]. HLA-A alleles are also associated with Guillain-Barré syndrome (GBS) in different populations [103,104,105]. GBS is a rare neurological disorder in which the body’s immune system attacks part of its peripheral nervous system [106]. In 2020, the HLA-A33 allele was found in a SARS-CoV2 induced GBS patient [107]. HLA-A also is associated with acute inflammatory demyelinating polyradiculoneuropathy (AIDP) [108]. Since limited research on HLA-A’s function in nervous system pathologies exist, contrasting associations have been shown between HLA-A and GBS [109,110]. In Iraqi patients with GBS, decreased HLA-A:0101 frequency was found in 2016 while in 2014, in GBS patients from East Coast of Australia, HLA ligands were found to be more prevalent [109,110]. Interactions between HLA-A and killer immunoglobulin-like receptors (KIRs) have been associated with GBS and Multiple Sclerosis (MS) patients as either risk or protective factors [109]. In 1998, HLA types were found to be associated with GBS onset in Japanese patients [111]. HLA-A role has also been shown to be important in Schwann cells that act as facultative APCs in peripheral nervous system and increase HLA Class I expression during GBS [112]. Therefore, additional work is warranted to understand the role of HLA-A in ZIKV infections. Finally, HSPA5 KD also increased ZIKV NS1 expression (Figure 10). Since HSPA5 interacts with the ZIKV envelope, regulated unfolded protein response, and alters the ER environment [83,113,114,115], its role in flaviviral infection also needs to be further examined.

Numerous proteomic and transcriptomic studies have been conducted to explain ZIKV infection in microcephalic fetuses, primary human fetal neural progenitor cells, serum samples, placental tissues, and astrocyte-derived cell lines [45,46,47,49]. This study identified numerous cellular proteins, some of which were similar to previous studies while others were novel. Among the similarities, fibronectin was found to be downregulated by ZIKV in U251 cells in this study. Our previous SOMAScan proteomic study also implicated ZIKV-induced damage to placental integrity [45,54]. Cytokines and chemokines such as IL-6 were identified as downregulated while IL-8 and CCL5 were upregulated in this study (Table 2) and in our previous study [54]. They were also found to be upregulated in ZIKV-infected human brain cortical astrocytes [116]. RNA-seq studies done in mouse primary astrocytes revealed common functions such as neuron development, brain development and neuromuscular diseases to be dysregulated by ZIKV [53], similar to the current study (Figure 3). Insulin like growth factor responses were also implicated in ZIKV infection by Shereen et al. [53], highlighting the potential role of IGFBP5 in ZIKV infection. Therefore, further studies to explore it are warranted. EDNRB also was one of the genes that was identified as down-regulated at both the mRNA and protein levels, respectively [53] and at the protein level in the current study (Table 2). An orthogonal study in 2018 on ZIKV infection in human neural progenitor neuronal cell line SK-N-BE2 also identified markers involved in similar cellular functions and processes as were identified in this study, including cell growth, cell cycle, cell death, NS development and function, and neurological diseases (Figure 2 and Figure 3) [117]. In addition, molecules involved in PI3K/AKT and ERK/MAPK pathways were found to be implicated in ZIKV infection in both this study (Figure 2 and Figure 3) and the orthogonal study [54,117]. In 2021, a TMT 10-plex system approach followed by LC-MS/MS on 12 placental samples from 2016 in Puerto Rico revealed cell–cell signaling and neurological disease as among the top dysregulated pathways and functions [46], similar to some of the findings in our study (Figure 2 and Figure 3). In addition to similarities, there are a few differences between this study and the previously published omics studies. For example, we used human glioblastoma astrocytoma U251 cells as a model cell line while Scaturro et al., 2018 used human neural progenitor cells, Shereen et al., 2021 used mouse primary astrocytes and Borges-Vélez et al., 2021 used placental samples [46,53,117]. Furthermore, Shereen et al. performed an RNA-seq study while we complemented the LC-MS/MS proteomic study with genetic KD [53]. Among the novelties in the findings of the current study, antigen presentation was predicted to be upregulated by ZIKV infection in U251 cells at the protein level (Figure 3) [49]. This is consistent with the increase in HLA-A expression observed in ZIKV-infected U251 cells and the consequent increase in ZIKV replication and NS1 protein synthesis post HLA-A KD conditions (Table 2; Figure 10). Energy production dysregulation was a cellular function differentially identified in this study unlike LC-MS/MS studies done previously on primary neural progenitor cells and human placental samples in 2018 and 2021 [46,49]. Another difference between this study and the orthogonal study done by Scaturro et al. is that they focused on ZIKV host protein–protein interactions and phosphoproteomic profiling via affinity purification integrated LC-MS/MS (AP-LC-MS/MS) and on kinase substrate relations/regulatory sites through PhosphitePlus51 resource while this study looked at proteomic impact of ZIKV infection via LC-MS/MS.

## 5. Conclusions

In conclusion, ZIKV dysregulates U251 cellular networks, functions and canonical pathways at the cell-wide level, with the impact being more severe at later time points than at earlier time points. The siRNA-mediated KD revealed that proteins like APOBEC3D, HLA-A and IGFBP5 KD increase ZIKV titers while TIMP2, MBD4 and TNC KD decrease ZIKV titers by either 48 or 72 hpi. Further analyses of ZIKV NS1 and ZIKV titers revealed that PSMA2, HSPA5, HLA-A and IGFBP5 behave as restriction factors for ZIKV replication in U251 cells by 48 hpi. Finally, ZIKV restores the expression of HLA-A in HLA-A KD U251 cells by 48 hpi; therefore, further experiments need to be conducted to better understand ZIKV infection. Moreover, the use of potential pharmaceutical compounds like MG132, a PSMA2 inhibitor known to exert anti-viral activity against HSV-1, trematinib which increases HLA-A expression via IFN gamma/STAT1 signaling and STAT3 activation and other tyrosine kinase inhibitors, need to be employed to identify novel anti-viral compounds against ZIKV infection [118,119,120,121]. The similarities and novelties of the current study in comparison to previously published omics studies help us understand some of the findings from this study better and further experiments need to be conducted to explain some of these differences and similarities across different cell types and omics platforms.

## Figures and Tables

**Table 1:** Number of significantly dysregulated proteins ^1^ in ZIKV-infected U251 cells.

# Significant ^2^	Time Post ZIKV Infection		
	12 h	24 h	48 h
	157 ^3^	681 ^3^	1949 ^3^
F.C. > 1.000	78	139	910
F.C. < 1.000	79	542	1039
F.C. > 1.100	74	135	757
F.C. < 0.9091	65	480	901
F.C. > 1.250	32	104	205
F.C. < 0.800	15	60	150
F.C. > 1.333	24	89	135
F.C. < 0.750	9	21	65
F.C. > 1.500	14	65	85
F.C. < 0.667	5	8	21
F.C. > 1.750	7	39	49
F.C. < 0.5714	3	4	5
F.C. > 2.000	5	23	33
F.C. < 0.500	1	1	2
F.C. > 2.500	0	11	16
F.C. < 0.400	0	1	1

^1^ The dysregulated proteins are found by mass spectrometric analyses of ZIKV infected U251 compared to time-matched mock-infected U251 cells. ^2^ Significance determined by two-tailed Students *t*-test and Z-score analyses. ^3^ These values are the total number of statistically significantly dysregulated proteins in U251 cells at each time point due to ZIKV infection, with no fold-change cut-off applied. # Significant means number of significant proteins.

**Table 2:** Number of significantly downregulated and upregulated targets in ZIKV-infected U251 cells at 24 and 48 hpi ^1,2^.

		24 h		48 h	
Gene Symbol	Name	*p*-Value	Fold Change	*p*-Value	Fold Change
EDNRB	Endothelin B receptor	- ^3^	- ^3^	0.0003	−2.82
CXCL14	C-X-C motif chemokine 14	0.0057	−1.84	0.0000	−2.06
APOBEC3D	DNA dC- > dU editing enzyme APOBEC-3D	0.0453	−3.48	- ^3^	- ^3^
STC1	Stanniocalcin-1	0.0024	−2.75	0.0002	−14.47
SFRP1	Secreted frizzled-related protein 1	0.0072	−1.78	0.0004	−7.29
IGFBP5	Insulin-like growth factor-binding protein 5	0.0058	−1.59	0.0000	−6.90
APP	Amyloid beta A4 protein	0.0028	−1.71	0.0002	−6.51
CFI	Complement factor I	0.0115	−1.53	0.0005	−5.89
HS6ST1	Heparan-sulfate 6-Osulfotransferase 1	0.0068	−1.41	0.0003	−5.11
GRN	Granulins	0.0039	−1.51	0.0002	−5.05
MDK	Midkine	0.0040	−1.70	0.0016	−5.02
FGFR1	Fibroblast growth factor receptor 1	0.0109	−1.54	0.0003	−4.99
LRIG3	Leucine-rich repeats and Immunoglobulin-like domains protein 3	0.0085	−1.57	0.0002	−4.92
IGFBP2	Insulin-like growth factor-binding protein 2	0.0010	−1.86	0.0000	−4.70
PCSK9	Proprotein convertase subtilisin/kexin type 9	0.0170	−1.53	0.0000	−4.53
MFGE8	Lactoadherin	0.0030	−1.70	0.0010	−4.37
CST3	Cystatin-C	0.0010	−2.07	0.0010	−4.31
IGFBP7	Insulin-like growth factor-binding protein 7	0.0100	−1.64	0.0006	−4.23
C4A	Complement C4b	0.7106	1.04	0.0042	−4.07
PLAU	Urokinase-type plasminogen activator	0.0408	−1.28	0.0007	−3.95
TNC	Tenascin	0.0101	−1.37	0.0004	−3.90
GPNMB	Transmembrane glycoprotein NMB	0.4192	−1.09	0.0018	−3.88
FSTL1	Follistatin-related protein 1	0.0104	−1.52	0.0005	−3.82
GFRA1	GDNF family receptor alpha-1	0.0029	−1.34	0.0008	−3.75
LAMA1	Laminin	0.0195	−1.31	0.0015	−3.61
CTSA	Lysosomal protective protein	0.0046	−1.57	0.0006	−3.56
THBS2	Thrombospondin-2	0.0162	−1.41	0.0008	−3.45
ACVR1B	Activin receptor type-1B	0.1943	1.14	0.0018	−3.41
XRCC6	X-ray repair cross-complementing protein 16	0.3570	−1.10	0.0004	−3.41
GNS	N-acetyl glucosamine-6-sulfatase	0.1788	−1.14	0.0006	−3.24
CCL13	C-C motif chemokine 13	0.3310	1.08	0.0014	−3.07
TIMP2	Metaloproteinase inhibitor 2	0.0044	−1.52	0.0003	−3.05
CCL20	C-C motif chemokine 20	0.4211	1.10	0.0032	−3.05
LRRC17	Leucine-rich repeat containing protein 17	0.0008	8.88	- ^3^	- ^3^
ISG15	Ubiquitin-like protein ISG15	0.0002	4.40	0.0001	2.74
HLA-A	HLA Class I histocompatibility antigen, A-33 alpha chain	0.0001	3.81	- ^3^	- ^3^
OAS2	2’-5’-oligoadenylate synthase 2	0.0001	3.46	0.0001	3.87
DHX58	Probable ATP-dependent RNA helicase DHX58	0.0005	3.17	0.0005	2.36
MX2	Interferon-induced GTP-binding protein MX2	0.0001	3.14	0.0000	2.51
IFIT2	Interferon-induced protein with tetratricopeptide repeats 2	0.0004	2.78	0.0004	2.52
MBD4	Methyl-CpG-binding domain protein 4	0.0005	2.74	0.8478	1.01
IFI44	Interferon-induced protein 44	0.0001	2.73	0.0003	1.68
DMXL2	DmX-like protein 2	0.0008	2.67	0.0020	1.70
IFIT3	Interferon-induced protein with tetratricopeptide repeats 3	0.0002	2.59	0.0002	2.47
HLA-B	Major histocompatibility complex, Class I, B	0.0002	2.37	0.0000	4.20
IFIT1	Interferon-induced protein with tetratricopeptide repeats 1	0.0002	2.35	0.0001	3.03
CXCL11	C-X-C motif chemokine 11	0.0050	2.76	0.0000	8.61
CCL5	C-C motif chemokine 5	0.0320	1.32	0.0000	4.72
CKB	Creatine kinase B-type	0.9310	1.01	0.0010	3.85
SSRP1	FACT complex subunit SSRP1	0.0110	1.42	0.0000	2.95

^1^ Cut-off *p*-value is 0.05 and the fold change threshold for ZIKV-infected U251 samples is kept at +/− 2.5 times that of time-matched mock-infected samples. *p*-value is calculated using 2-tailed Students T-test comparing ZIKV-infected U251 samples to time-matched mock-infected U251 samples. ^2^ Positive fold-change values indicate protein quantities higher in ZIKV-infected samples than in mock-infected samples. ^3^ indicates proteins was not detected by mass spectrometry at indicated time point.

## Data Availability

Not applicable.
